# Estrogen receptor signaling regulates the expression of the breast tumor kinase in breast cancer cells

**DOI:** 10.1186/s12885-018-5186-8

**Published:** 2019-01-16

**Authors:** Sayem Miah, Edward Bagu, Raghuveera Goel, Yetunde Ogunbolude, Chenlu Dai, Alison Ward, Frederick S. Vizeacoumar, Gerald Davies, Franco J. Vizeacoumar, Deborah Anderson, Kiven Erique Lukong

**Affiliations:** 1 Department of Biochemistry, Microbiology & Immunology, College of Medicine, University of Saskatchewan, 107 Wiggins Road, Saskatoon, SK S7N 5E5 Canada; 2 Cancer Research, Saskatchewan Cancer Agency, and Division of Oncology, College of Medicine, University of Saskatchewan, Saskatoon, SK S7N 5E5 Canada; 3 Department of Anatomy and Cell Biology, University of Saskatchewan, Saskatoon, Saskatchewan Canada; 4 Department of Pathology, University of Saskatchewan, Saskatoon, S7N 0W8 Canada; 5 College of Pharmacy and Nutrition, University of Saskatchewan, Saskatoon, S7N 5C9 Canada

**Keywords:** BRK, Tamoxifen, Estrogen receptor, Breast cancer, Tyrosine kinase

## Abstract

**Background:**

BRK is, a non-receptor tyrosine kinase, overexpressed in approximately 85% of human invasive ductal breast tumors. It is not clear whether BRK expression correlates with breast cancer subtypes, or the expression has prognostic or diagnostic significance. Herein, we investigated the correlation of BRK with any breast cancer subtypes and clinicopathological significance of BRK expression in breast cancer.

**Methods:**

In this study, we examined BRK expression in 120 breast tumor samples and 29 breast cancer cell lines to explore the positive correlation between BRK and the expression of ERα. We used immunohistochemistry, RT-PCR, and immunoblotting to analyse our experimental samples.

**Result:**

We demonstrate that estrogen induces *BRK* gene and protein expression in ER+ breast cancer cells. Over-expression of ERα in the ER-negative breast cancer cell line increased BRK expression, and knock-down of ESR1 in MCF7 cells reduced BRK levels. Further, we provide evidence that BRK is regulated by ERα signaling and the presence of ER antagonists (tamoxifen and fulvestrant) reduce the expression of BRK in ER-positive breast cancer cells. Finally, we demonstrate that the overall survival of ER-positive breast cancer patients is poor when their cancers express high levels of BRK.

**Conclusion:**

Our data indicate that BRK is a prognostic marker for ER+ breast cancers and provide a strong rationale for targeting BRK to improve patients’ survival.

**Electronic supplementary material:**

The online version of this article (10.1186/s12885-018-5186-8) contains supplementary material, which is available to authorized users.

## Background

Breast tumors are classified into four major molecular subtypes [1–3]: HER2 (human epidermal growth factor receptor 2) type, Basal cell type, and Luminal A and B types. The HER2 type displays an overexpression of HER2, is usually high-grade and occurs in about 25% of cases [4]. The Basal cell type is mostly characterized as triple-negative breast cancer (TNBC) because of the lack of ER and progesterone receptor (PR) expression and HER2 amplification. This subtype has a high proliferation rate, has a poor prognosis and occurs in about 5–10% of breast cancer patients [5]. The Luminal A and B types are ER-positive and occur in nearly 75% of breast tumors [1–3, 6]. The ER signaling pathway plays a critical role in mammary gland development and is activated by its ligand, estrogen or estradiol (E2) [7]. The ER is, therefore, a prime therapeutic target for luminal breast cancers. The ER is targeted directly by antiestrogen agents such as the partial antagonist tamoxifen (Tam) and pure antagonist fulvestrant [8], and indirectly by aromatase inhibitors (AIs) that block the production of estrogen [9].

BRK is overexpressed in approximately over 85% of breast carcinomas, but low or undetected in the normal mammary gland [10]. BRK has been implicated in several signaling cascades, especially mitogenic signaling [11]. Recently, we showed that BRK activation significantly enhanced tumor formation in xenograft models [12]. Targeted overexpression of BRK in the mouse mammary gland was shown to enhance survival of the mammary epithelial cells and tumor formation and induce delayed involution [13, 14]. Surprisingly, no investigation has been undertaken to explore whether the overexpression of BRK is linked with any of these major subtypes.

Given the high expression of BRK in the majority of breast cancers reported, we set out to investigate whether there was a functional link between BRK expression and the various molecular subtypes of breast cancer. We found a correlation between BRK expression and ER expression in ER-positive breast cancers. We, therefore, explored the functional link between BRK and ERα signaling in ER-positive breast cancer. Additionally, the clinical relevance of BRK expression in tumors of ER-positive breast cancer patients was investigated.

## Methods

### Cell culture

All breast cancer cell lines were obtained from the American Type Culture Collection (ATCC, Manassas, Virginia, USA). They included AU565, BT20, BT474, BT549, HCC38, HCC70, HCC1187, HCC1395, HCC1419, HCC1428, HCC1569, HCC1599, HCC1806, HCC1937, HCC1954, Hs578T, MCF7, MDA-MB-134, MDA-MB-175, MDA-MB-231, MDA-MB-361, MDA-MB-468, MDAkb2, SKBR3, T47D, UACC812, and UACC893. MCF10A and MCF12cell lines, derived from normal human breast epithelia were also obtained from ATCC. Both sets of cell lines were obtained as part of the ATCC Breast Cancer Cell Panel (ATCC® 304500 K™) and were cultured according to ATCC recommendations for fewer than six months from the time of resuscitation. The supplier performed authentication.

### RNA isolation, reverse transcription, PCR and quantitative real-time PCR (qPCR)

Total RNA was isolated from all the cell lines using TRizol as was recommended by the manufacturer (Invitrogen). The RNA quantity and quality were analyzed using a spectrophotometer and gel electrophoresis, 1.5 μg of total RNA was then used as a template to generate 20 μL, of first strand cDNA using the Thermo-scientific maxima first strand cDNA synthesis Kit as was recommended by the manufacturer (#K1642; Thermo Scientific). The procedure was carried out as we previously described [15].

### Immunohistochemistry and density analysis

Immunohistochemistry (IHC) staining on human breast cancer tissue array BR10010a and BR243d with rabbit anti-BRK (C-18) antibody was performed and analyzed by USBIOMAX (https://www.biomax.us/). Briefly, the tissue samples on each array were formalin fixed, paraffin embedded. Tissue array sections were mounted on the positive charged SuperFrost Plus glass slide. Primary antibody rabbit anti-BRK(C-18) antibody (sc-1188) was purchased from Santa Cruz Biotechnology, Inc. ImmPRESS™ Reagent anti-Rabbit Ig (peroxidase) of catalog number MP7401 were purchased from Vector Laboratories. DAB (DAKO Cytomation, Code K3465) used as substrate chromogen. Antigen retrieval solution was purchased from DakoCytomation (Target Retrieval solution, S-1699). The standard procedure can be obtained through https://www.biomax.us/.

### Immunoblotting

Proteins derived from either whole cell lysates or derived from immunoprecipitations were resolved via SDS-PAGE in 10% polyacrylamide gels. The resolved proteins were then transferred onto nitrocellulose membranes and immunoblotted with indicated antibodies as previously described [16].

### Luciferase assays

Cells were co-transfected with the BRK promoter (− 964 to + 1; 250 ng/well) along with an effector plasmid that encodes for either the ESR1 or ESR2 full-length protein [17]. Twelve hours after transfection, cells were then treated 17β-estradiol (10 μM) for 24 h and Luciferase activities determined on the TD-20/20 Luminometer (Turner Designs). Using the LightSwitch Assay Reagent (Active Motif, Carlsbad) as recommended by the manufacturer to determine.

### Transfection and stable cell line preparation

The GFP-BRK constructs were generated as described before [12]. Plasmids encoding GFP-ERα and GFP-ERβ were a kind gift of Dr. Michael Mancini (Baylor College of Medicine, Houston, Texas 77,030, USA). Plasmids encoding the BRK shRNA and ER shRNA sequences were procured from Santa Cruz Biotechnologies (sc-29,305-SH and sc-108,060, respectively, Santa Cruz, CA USA). All plasmids used were transfected in the indicated cell lines using polyethyleneamine (PEI) (23966–2, Polysciences Inc., PA, USA) as the transfection reagent. Cells were seeded in 10 cm dishes and cultured to approx. 70–80% confluency before transfection. Briefly, 10 μg plasmid DNA was first diluted in 430 μl 0.15 M NaCl via gentle vortexing. Next, 60 μl PEI was added and the mix vortexed briefly. The transfection mix was incubated at room temperature for 10 min to allow the formation of DNA-PEI complexes and was then dispensed drop-wise into the culture dishes. The dishes were then swirled gently to allow even distribution of the DNA-PEI complexes and incubated at 37 °C overnight. After 24–48 h post transfection, where necessary, the transfection efficiency was assessed via visualizing the cells on a fluorescent microscope (1 × 51 Olympus X-cite series, ON, CA) before proceeding with further experiments.

For the generation of a stable BRK or ER knockdown cell line, MCF7 cells were transfected as described above with shRNA-carrying plasmids targeting either the BRK or ER message. 24 h post-transfection, cells stably incorporating the shRNA sequences were selected with 2.5 μg/mL puromycin and cultured to confluency. BRK and ER knockdown was then verified via Western blotting using the appropriate antibodies. The established stable cell lines were maintained under a minimal puromycin dose (0.5 μg/mL) [12].

### Estradiol, tamoxifen and fulvestrant treatment

Cultured cells were treated with varying doses of estradiol (E2) (10,006,315, Cayman Chemicals) to determine the optimal working concentration. The cells were seeded in 6-well plates, and cultured in media supplemented with either 20, 10, 1, 0.1, 0.001 μM E2 for 24 h. Dimethylsulfoxide (DMSO) and tamoxifen (4-hydroxytamoxifen) were purchased from Sigma Chemical Co. (USA). Fulvestrant was purchased from Cayman Chemical (USA). Cells were treated at an indicated concentration of the antagonists and cell lysates analyzed by immunoblotting.

### In silico analysis of BRK expression from the RNA-seq data

RNA-seq Version 2 data containing tumor samples of 24 different types of cancer and adjacent non-tumor tissues were downloaded from The Cancer Genome Atlas project (TCGA) website (https://cancergenome.nih.gov/). The TCGA dataset itself is publicly available and contains multi-dimensional maps of the key genomic changes in 33 types of cancer from more than 11,000 patients. We used only the RNA-seq Version 2 dataset for analysis of BRK gene expression.

### Analyses of outcome for overall survival and relapse-free survival

To evaluate the relationship between BRK expression and the patient clinical outcome we used the KM Plotter Online Tool (http://kmplot.com/analysis/) in different breast cancer subtypes [18]. This is a public database contained information from 5143 breast patients that permits to investigate the association of genes with overall survival (OS) and relapse-free survival (RFS).

### Statistical analysis

One-way ANOVA followed by a post hoc Newman-Keuls test was used for multiple comparisons using GraphPad Prism version 5.04 for Windows, GraphPad Software, San Diego California USA, https://www.graphpad.com/. Spearman correlations were determined as described by the developer (https://www.wessa.net/rwasp_spearman.wasp/). Significance was set at *P* < 0.05 and error reported as plus or minus the standard deviation.

## Results

### BRK mRNA is overexpressed in most human tumors

**Fig. 1:**
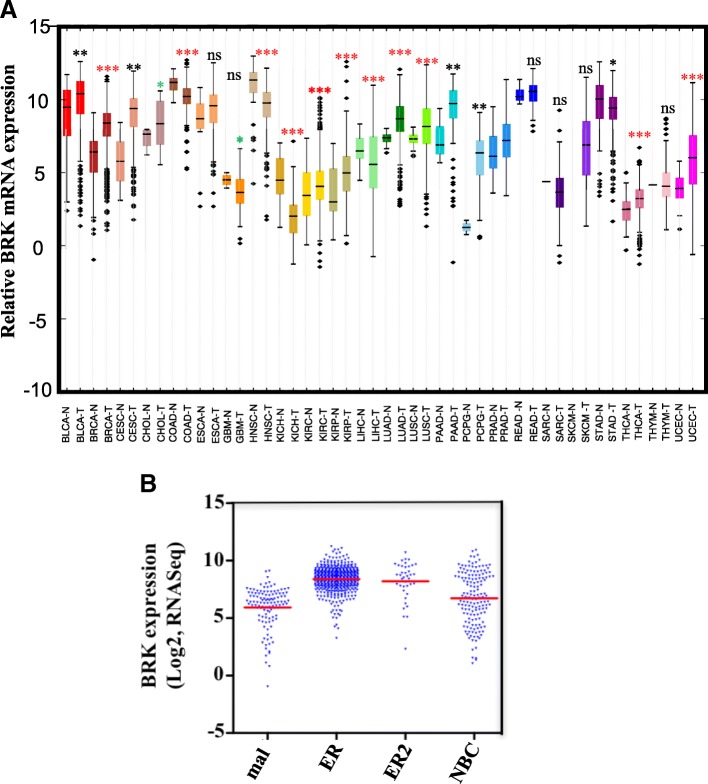
BRK is overexpressed in most human tumors. **a** Differential expression data of *BRK* mRNA between normal and tumor tissue for 24 human cancers. Data obtained from The Cancer Genome Atlas database, median ± one quartile; *p < 0.05; **p < 0.01; ***p < 0.001; ns = not significant (also see Additional file 1: Table S1 for details). Tissue samples are denoted N for normal and T for tumor. BLCA = bladder urothelial carcinoma, BRCA = breast carcinoma, CESC = cervical squamous cell carcinoma, CHOL = cholangiocarcinoma, COAD = colon adenocarcinoma1, ESCA = esophageal cancer, GBM = glioblastoma multiforme, HNSC = head and neck squamous cell carcinoma, KICH = chromophobe renal cell carcinoma, KIRC = clear cell kidney carcinoma, KIRP = papillary kidney carcinoma, LIHC = liver hepatocellular carcinoma, LUAD = lung adenocarcinoma, LUSC = lung squamous cell carcinoma, PAAD = pancreatic ductal carcinoma, PCPG = pheochromocytoma and paraganglioma, PRAD = prostate adenocarcinoma, READ = rectal adenocarcinoma, SARC = sarcoma, SKCM = cutaneous melanoma, STAD = stomach adenocarcinoma, THCA = papillary thyroid carcinoma, THYM = thymoma, UCEC = uterine corpus endometrial carcinoma. **b** BRK transcript levels are significantly higher in ER-positive breast cancers. Data on *BRK* gene expression mined from The Cancer Genome Atlas (TCGA) database. Analyses of TCGA data were performed on breast tissue samples with RNA-sequencing data. Log2 transformed data was obtained from normal mammary tissue samples (*n* = 114) and from a total of 683 breast cancers classified as ER-positive (*n* = 492), HER2-postive (*n* = 39), and TNBC (*n* = 152). Statistical significance was calculated against the normal tissue: *p*-value 8.1 × 10^− 45^ (ER-positive); p-value 2.3 × 10^− 11^ (HER2-postive); *p*-value 0.002 (TNBC). *P* < 0.005 = significant

BRK is overexpressed in breast carcinomas and has also been detected at elevated levels in a few other cancer types [16, 19–21]. We compared the expression pattern of BRK mRNA in 24 different cancer types provided by TCGA (https://cancergenome.nih.gov/). We observed that *BRK* mRNA expression was higher in most of the cancers compared to the non-cancerous tissues (Fig. 1a). Fifteen of 24 cancer showed expression levels that were significantly higher (*P* < 0.05) than their respective normal tissues. Six different cancer types displayed lower levels of *BRK* mRNA compared to normal tissue, whereas three cancer types had too few samples to determine statistical significance (Additional file 1: Table S1). The most significant difference (*P* = 1.2 × 10^− 31^) was observed in the breast cancer cohort, comprising of 100 normal mammary tissues and 1084 breast carcinoma tissue samples (Additional file 1: Table S1). Our findings indicate that BRK mRNA is upregulated in most cancers, but the differential expression of BRK is most significant in breast cancer as compared to normal tissues.

### BRK transcript levels are significantly higher in ER-positive breast cancers

Next, we cross-examined a breast carcinoma (BRCA) cohort and stratified samples into the three main breast cancer subtypes: HER2+, ER+ and to TNBC, for relative differences in the transcript levels. As it is shown in Fig. 1b, the log2 fold change of the *BRK* mRNA in different subtypes of breast cancers. It demonstrated significantly higher expression of mRNA in luminal (ER+) breast cancers (*P* = 8.1 × 10^− 45^) compared to HER2-positive or TNBC subtypes, with a *P* values of 2.3 × 10^− 11^ and 0.002, respectively (Additional file 1: Table S2**)**. Both the total intensities and a number of positives were higher in the ER-positive samples compared to other subtypes (Additional file 2: Figure S1). These data demonstrate that although *BRK* mRNA is upregulated in all breast cancer subtypes; this increased expression is more enhanced in ER-positive breast cancers.

### BRK protein expression correlates with tumor progression

**Fig. 2:**
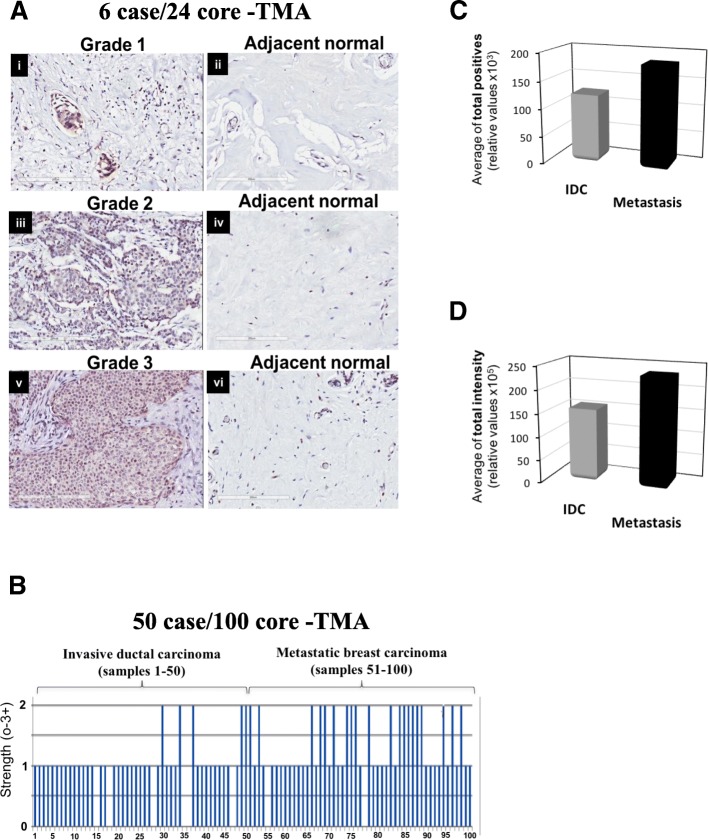
Immunoreactivity of BRK increased significantly with tumor grade and stage. **a** BRK expression was determined via immunohistochemistry (IHC) analyses on a 6 cases/24 cores breast cancer tissue microarray (TMA) (BR243d, USBIOMAX, USA) with matched adjacent normal breast tissue, and includes information about TNM, clinical stage and pathology grade. **b** BRK expression was determined by IHC analyses on a breast cancer tissue array, BR10010a (USBIOMAX, USA). The TMA contained a 50 cases/100 cores array containing 50 invasive carcinoma samples and matched lymph node metastasis samples. BRK expression increases with metastatic index. Samples 1–50 and samples 51–100 show the strength of BRK expression in 0–3+ scale in invasive ductal carcinoma and in metastatic breast carcinoma tissue, respectively. Anti-BRK antibody was used to stain the tissue sections and specific binding was detected with ImmPRESS TM reagent followed by color development in peroxidase substrate DAB (3,3′-diaminobenzidine). **c** Represents the average of the total number of samples that stained positively for BRK in all IDC samples versus metastatic carcinoma samples, and **d** the average total intensity for BRK in all IDC samples versus metastatic carcinoma samples

To determine whether the observed differential expression pattern of *BRK* mRNA in breast cancer subtypes is corroborated at the protein level, we first examined the expression of BRK in tissue microarrays (TMAs). Two TMAs (US Biomax, MD, USA) were used in the study. The first TMA is a 6 cases/24 cores array that contains 12 invasive ductal carcinomas (IDC) samples, classified according to tumor grade, and 12 adjacent normal mammary tissues (Additional file 1: Table S3). The second TMA (50 cases/100 cores) contained 50 cases of breast carcinoma and 50 matched lymph node metastasis (LNM) samples (Additional file 1: Table S4). Tissue staining intensities for BRK were scored using a 4-point scale 0–3+, where 0 = no staining, 1 = low staining, 2 = moderate staining, and 3 = strong staining. Analysis of the 6 case/24 core-TMA (Additional file 1: Table S3) revealed that: 1) BRK was overexpressed in the tumors, but low or absent in the adjacent normal tissues in all samples (Fig. 2a)**;** and 2) BRK immunoreactivity increased significantly with tumor grade with the lowest expression in Grade 1 and the highest staining in Grade 3, whereas Grade 2 displayed an intermediate level of expression of BRK (Fig. 2a).

Next, we analyzed the differential expression of BRK in IDC and metastatic carcinoma samples in the 50 case/100 core-TMA (Additional file 1: Table S4). We observed BRK immunoreactivity in over 80% of specimens, with staining score of + 1 or + 2 (Fig. 2b). Only 5 out of 50 (10%) IDC samples had + 2 intensity (Fig. 2b). More strikingly, 20 out of 50 (40%) metastatic carcinoma samples displayed a 2+ BRK staining (Fig. 2b). The total number of BRK-positive samples were distinctly higher in the metastatic carcinoma samples compared with the IDC samples (Fig. 2c and d).

### BRK protein expression is generally higher in ER-positive breast carcinomas compared to other subtypes

**Fig. 3:**
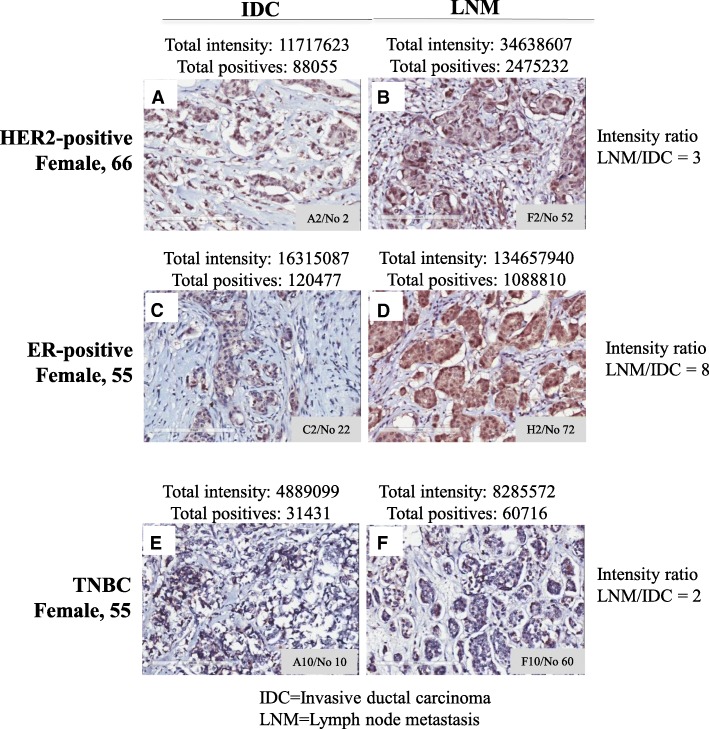
BRK staining in invasive and lymph node metastatic carcinomas show higher expression in ER-positive tumors. Representative immunohistochemical staining of BRK (**a-f**) in human breast TMA. tumor samples selected form a 50 cases/100 cores array (BR10010a, USBIOMAX, USA). The TMA was a 50 cases/100 cores array containing 50 invasive carcinoma samples and matched 50 lymph node metastasis samples. The age and clinical details of each case including TNM and pathology grade, ER, PR and HER2 status were available (http://www.biomax.us/tissue-arrays/Breast/BR10010a). TNBC cases were those that were negative for ER, PR and HER2. The absolute values for the total intensity and total number of positive BRK staining were provided by the pathologists at USBIOMAX. LNM = lymph node metastasis, IDC = invasive ductal carcinoma. The Intensity ratio was calculated as Total intensity values of LNM over IDC. A2/No 2, F2/No 52, c2/No 22, H2/No 72, A10/No 10, and F10/No 60 designations, located at the lower hand corner of each sample, represent the position of the sample on the BR10010a (http://www.biomax.us/tissue-arrays/Breast/BR10010a)

We next compared the values from the IDC and the lymph metastasis (LNM) in the TMAs. Representative IHC data are shown in Fig. 3. We detected the strongest expression of BRK in samples derived from ER-positive patients, compared with HER2, PR alone or TNBC samples (Fig. 3c and d). It is worth noting that the intensity of BRK expression in ER-positive LNM samples is 16-fold higher than the TNBC value. Further, we noted that for each patient, the expression of BRK (total intensities and a total number of positives) increased as cancer progressed from IDC to LNM (for example, compare Fig. 3c and d values). The LNM to IDC ratio for total intensity was higher in ER-positive patients compared to the ratio in HER2-positive and TNBC patients (Fig. 3): ratio was 8, 3, and 2 respectively for ER-positive (Fig. 3c vs d), HER2-positive (Fig. 3a vs b) and TNBC (Fig. 3e vs f) samples. These results demonstrate that: 1) the expression of BRK protein increases with malignancy even within the same patient, and 2) the expression of the protein is higher in ER-positive samples compared to the other subtypes. Our findings suggest that BRK is a marker for ER-positive breast tumor progression in particular.

### BRK protein and transcript levels are elevated in ERα + breast cancer cells

**Fig. 4:**
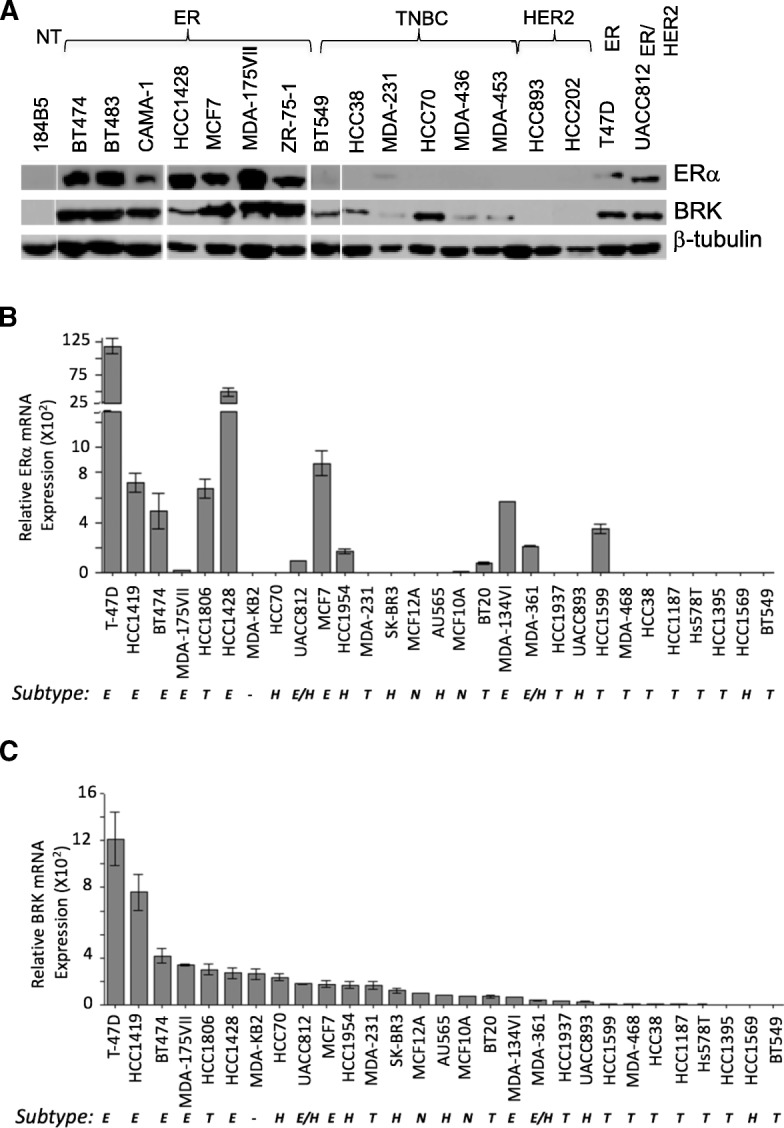
BRK protein and transcript levels are elevated in ER-positive breast cancer cells. **a** Lysates from 184B5, a transformed cell line established from normal mammary tissue, and 17 breast cancer cell lines, with cell lines subtype as defined by Neve and colleagues Neve et al. [22], were evaluated for BRK and ERα expression by immunoblotting. β-tubulin was used as the loading control. **b** and **c** *BRK* and *ERα* mRNA expression was assessed by quantitative reverse transcriptase PCR (qPCR). NT = normal transformed, E = ER-positive, T = triple negative, H=HER2, N = normal

Our IHC data demonstrate that the expression of BRK varies based on tumor grade and malignancy, as well as molecular class, and higher levels are observed in ER-positive tumors (Fig. 3). Like primary tumors, there is substantial variability amongst breast cancer cell lines based on some criteria including gene expression profile, molecular class, subtype, tumorigenicity and metastasis [22–26] (Additional file 1: Table S5). We examined the expression of BRK in a panel of 18 mammary epithelial cells. This panel included eight ERα-positive cell lines, six TNBC cell lines, and two HER2-positives, and one ER/HER2-positive cell line, as well as an immortalized, non-tumorigenic cell line (184B5) used as a control cell line. BRK protein expression was not detected in the control cell line, 184B5. As it is shown in Fig. 4a, all ERα-positive breast cancer cell lysates analyzed displayed high BRK expression. However, BRK expression level in the TNBC and HER2-positive cell lysate were low or undetected. These data, which suggested a correlation between the protein expression of BRK and ERα, also correlated with transcripts expression (Fig. 4b, c). Statistical analysis (Pearson Correlation) revealed a strong correlation (*R* = 0.77 and *P*-value = 0.000001) between BRK and ERα mRNA expression. Together, our data further provide evidence that there is a positive correlation between BRK expression and ERα status in breast cancers.

### 17-β-estradiol treatment induces BRK gene and protein expression

**Fig. 5:**
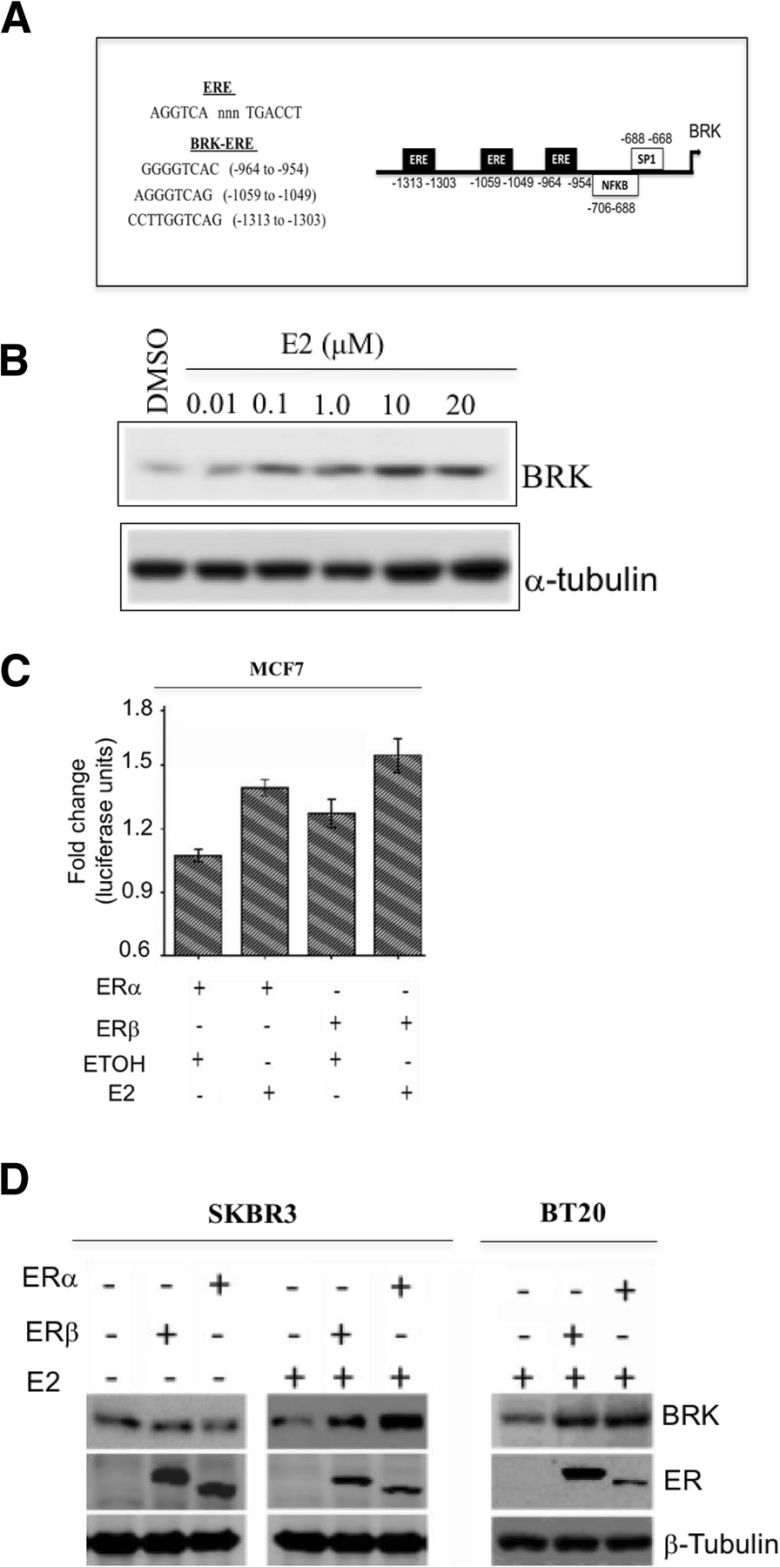
17-β-estradiol treatment induces BRK gene and protein expression. **a** Schematic of the *BRK* promoter showing the positions of three EREs and binding sites for SP1 and NFκ-B. **b** MCF7 cells were treated with increasing concentrations of 17-β-estradiol (E2) for 24 h. Cellular proteins were detected in total cell lysates by immunoblotting analysis with anti-BRK and anti-ERα antibodies and β-actin expression served as loading control. **c** Luciferase activity assay of the reporter constructs in MCF7 cells transfected with and without ERα and ERβ and with and without E2 treatment shows E2 responsive transcriptional activation of the BRK promoter. ETOH (ethanol) was used as a vehicle control. **d** Plasmids expressing ERα or ERβ were transiently transfected into ER-negative breast cancer cell lines SKBR3 and BT20 and the cell lines treated with either E2 or with a DMSO. Cell lysates were analyzed by immunoblotting using antibodies against BRK and ER. The expression of β-tubulin was used as a loading control

Since we observed that ERα is overexpressing cells and tissue display correspondingly high expression of BRK (Fig. 4), we investigated whether there was a functional link between BRK and ERα. ERs signal via different pathways including the nuclear estrogen response element (ERE)-dependent or -independent pathways [7]. We performed in silico analyses on the 5` UTR of the BRK promoter using MatInspector (http://www.genomatix.de/) and identified three EREs within a 1500 bp region proximal to the transcription start site of the BRK promoter (Fig. 5a), indicating a potential regulation of BRK gene expression via an ERE-dependent pathway. We, therefore, hypothesized that BRK might be a target gene of ER signaling. Thus, we treated the cells with increasing concentrations of E2 for 24 h and observed that E2 treatment resulted in a dose-dependent increase in BRK levels in the MCF7 cells (Fig. 5b). The same observation was made for ER-positive T47D cell line (Additional file 2: Figure S2). E2 treatment of ER-negative BT20 cells did not affect BRK levels (Additional file 2: Figure S2). However, E2 treatment induced the expression of the luciferase reporter in both the ERα- and ERβ-transfected cells (Fig. 5c). Further, ERα or ERβ overexpression in ER-negative cell lines, SKBR3 and BT20, induced upregulation of BRK protein levels. Taken together, our data indicate an E2-ER-mediated regulation of *BRK* and suggest a functional link between BRK and ER in ER-positive breast cancers.

### Loss of ERα and/or inhibition of ERα with tamoxifen and fulvestrant downregulates BRK expression in ER+ breast cancer cells

**Fig. 6:**
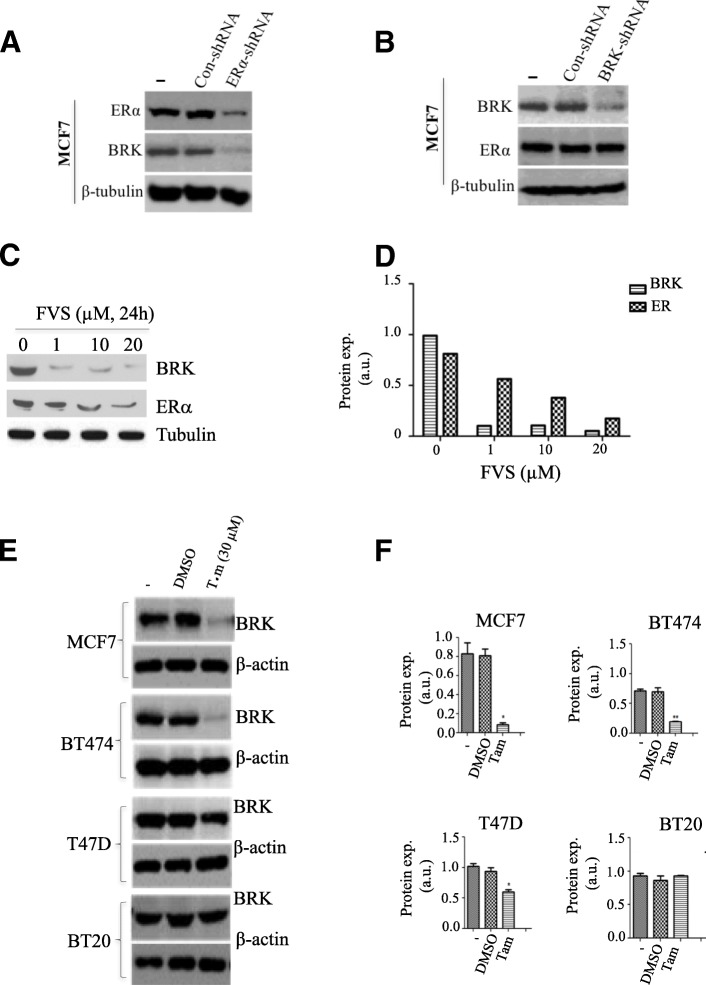
Loss of ERα and/or inhibition of ERα with tamoxifen and fulvestrant downregulate BRK expression in ER-positive breast cancer cells. **a**, **b** Lysate from MCF7 cells stably transfected with control shRNAs or shRNAs against ERα (**a**) or BRK (**b**) were analyzed by immunoblotting using antibodies against BRK and ERα, and β-tubulin as a loading control. **c** Immunoblot analysis of MCF7 cells treated with vehicle (DMSO) or increasing concentrations of fulvestrant (1–20 μM) for 24 h. **d** Quantification of immunoblots from (**c**). **e** Immunoblot analysis of MCF7, BT474, T47D and SKBR3 cells treated with vehicle (DMSO) or 4-hydroxytamoxifen (30 μM) for 24 h. **f** Quantification of immunoblots from (**e**). Protein expression was quantified using Image J software

Since E2 stimulation upregulated BRK mRNA and protein levels in ERα-positive breast cancer cell lines (Fig. 5), this implied that BRK was downstream of the ER-signaling pathway. We, therefore, investigated how the loss of ERα expression affects BRK protein expression. We knocked down ERα or the inhibited ER-signaling with ERα antagonists such as tamoxifen and fulvestrant to examine whether it suppresses BRK expression. As it is shown in Fig. 6a, we used ERα-shRNA to efficiently knock down ERα expression by approximately 75% in MCF7 cells, resulting in a dramatic decrease in the expression of BRK, by approximately 80% (Fig. 6). Notably, the reciprocal knockdown of BRK did not affect the expression levels of ERα (Fig. 6b). Further, we next tested if fulvestrant, an ERα down-regulator could modulate BRK expression and observed that fulvestrant treatment resulted in a dose-dependent reduction of BRK expression (Fig. 6c, d), which corresponded with the downregulation of ERα protein as reviewed previously [27]. Similar to fulvestrant, the effect of tamoxifen, a selective inhibitor of ERα function, was tested. We found that tamoxifen treatment resulted in a dose-dependent downregulation of BRK in ERα-positive cell lines MCF7, T47D, and BT474, but not in ER-negative BT20 cells (Fig. 6e, f). DMSO control did not affect BRK expression. Collectively, our data establish endogenous ERα as a positive regulator of BRK expression in breast cancer cells. Furthermore, we conclude that two clinically-relevant ERα antagonists with different mechanisms of action, fulvestrant, and tamoxifen, can be used to inhibit BRK expression.

### High BRK transcript level tends to correlative with ER+ breast cancer patient survival

In this study, we found that ERα regulates the expression of BRK in ER-positive breast cancer cell lines and tissues and that E2 signaling regulates BRK expression. Based on our observation, we hypothesized that BRK transcript expression might be associated with the clinical outcome of ER-positive breast cancer patients. Overall survival analysis of breast cancer patients’ samples from the TCGA data set revealed that: 1) ER-positive breast cancer patients have a better prognosis than all the other subtypes combined (*n* = 1102; *P =* 6.3e-07; Additional file 2: Figure S3A); 2) high *BRK* levels in the breast in breast cancer patients tend to be associated with shorter overall survival than patients with low *BRK* levels (n = 1102; *P* = 0.65; Additional file 2: Figure S3B); 3) high *BRK* levels in ER-positive tumors is associated with shorter overall survival, (*n =* 491*; P =* 0.84; Additional file 2: Figure S3C). We also identified an association between *BRK* expression and relapse-free survival (RFS) in ER-positive tumors as a whole (hazard ratio, HR = 1.14, *P* = 0.38) (Additional file 2: Figure S3D**),** and in ER-positive subtypes luminal A (HR = 1.2, *P* = 0.37) (Additional file 2: Figure S3E**)**, and luminal B (HR = 1.14, *P* = 0.13) (Additional file 2: Figure S3F**)** tumors. Although our data, in general, did not reach statistical significance, it can be deduced that high BRK expression was significantly associated with poor RFS in tamoxifen-treatment patients (*n* = 161; *P* = 0.0043; Additional file 2: Figure S3G), thus strengthening the rationale for ER/BRK co-targeting in ER-positive breast cancers.

## Discussion

Breast cancer is stratified into four main subtypes: the ER/PR+ luminal A and B subtypes, the HER2-positive subtype, and the TNBC, which is characterized by the absence ER and PR expression as well as the lack of HER2 overexpression or gene amplification. The luminal types comprise the most common group and occur in nearly 75% of breast tumors [1–3, 6]. In our study, we also found that BRK expression (mRNA and protein) correlates more with ER+ breast cancer tissue and cell lines. However, various studies in the past have correlated BRK expression with different breast cancer subtypes. Born et al. found a significant correlation between BRK and HER2 at the protein level [28]. Co-overexpression of BRK with HER3 and HER4 has also been reported [10]. Both studies suggested that BRK plays a functional role in the HER-signaling cascade. However, Irie et al. found a strong correlation between BRK expression and luminal B (ER+ and HER2+) breast cancers patients, but not with basal-like tumors [29]. We found that BRK is regulated by E2 signaling, supporting a functional link between BRK and ERα in ER-positive breast cancers. This mechanism of regulation in other subtypes is different. The expression of BRK is regulated by mitogenic signaling in HER2-positive breast cancers [28].

The E_2_–ER–ERE pathway plays a crucial role in regulating the oncogenic effect of the ER. We identified ERE sites in the BRK promoter and demonstrated that E2 stimulation resulted in increased levels of both BRK transcript and protein in ERα-positive breast cancer cells. Consequently, ERα-positive breast cancer cells treated with either tamoxifen or fulvestrant resulted in a dose-dependent decrease in BRK expression. However, ERα did not co-immunoprecipitate with the BRK promoter (data not shown). It could be reasoned that the BRK promoter region harbors critical cis-acting elements including those for transcription factors such as Sp1, AP1, and NF-kB [30], suggesting BRK gene expression via E2-signaling may be ERE-independent.

The prognostic significance of BRK in human malignancies is uncertain. Aubele et al. used a cohort of invasive breast cancer cases and demonstrated that the high BRK expression predicts low disease-free survival [31]. Publicly available gene expression microarrays data showed that ER-positive patients with high expression of *BRK* might be at increased risk of relapse **(**Additional file 2: Figure S3). We found that breast cancer patients with high levels of *BRK* mRNA, as well as ER-positive patients, tend to have worse overall survival probabilities, irrespective of the ER-positive subtypes. Irie et al. however used a different cohort and reported that the expression of BRK in Luminal B tumors, in particular, was associated with poor outcomes [29]. Additionally, our data also showed that in tamoxifen-treated patients high BRK is associated with poor RFS, which suggest that co-targeting ERα and BRK in ER-positive breast cancers are clinically relevant.

## Conclusions

In summary, our data show that BRK is overexpressed in most of the ERα-positive breast cancer cells and tissues. Additionally, we also found that ER regulates BRK expression in ER-positive cells and tissues by E2 signaling. Furthermore, we have observed that elevated BRK expression is unfavorable for the overall survival in ER-positive breast cancer patients. Therefore, based on the evidence presented we are proposing BRK as a potential ERα-associated co-biomarker that could be a combination therapeutic target for the treatment of ER+ breast cancer patients.

## Additional files

Additional file 1: **Table S1.** Differential expression of BRK mRNA in various cancers. **Table S2.** BRK mRNA expression in a TCGA cohort of breast cancer subtypes. **Table S3.** Invasive ductal carcinoma (IDC) samples, classified according to tumor grade. Clinical parameters for the 6 cases/24 cores array that contains 12 invasive ductal carcinoma (IDC) samples, classified according to tumor grade, and 12 adjacent normal mammary tissues. **Table S4.** Breast tumor samples classified according to lymph node metastasis ability. Clinical parameters for 50 cases/100 cores contained 50 cases of breast carcinoma (46 IDC, one micropapillary carcinoma, two invasive lobular carcinomas, and one neuroendocrine carcinoma) and 50 matched lymph node metastasis (LNM) samples. **Table S5.** Clinical and molecular characteristics of breast cancer and mammary epithelial cells. Classification of breast cancer cell lines as described by Neve et al. [22]. (PDF 404 kb)

Additional file 2: **Figure S1.** Molecular subtype of clinical tumor tissues. The absolute values for the total intensity and total number of positive BRK staining for each sample in the 50 cases/100 cores array (BR10010a, USBIOMAX, USA) were provided by the pathologists at USBIOMAX. Based on the clinical information provided, the samples were grouped into their respective molecular subtype: ER, PR, HER2, and triple negative. The average total intensities and number of positives for each subtype were calculated and plotted on the graphs. A) Average total intensity per subtype. B) Average total number of positive per subtype. **Figure S2.** Estradiol dose dependent BRK and ERα protein expression in breast cancer cell lines. MCF7, T47D and BT20 cells were treated with 0.001, 0.01, 0.1, 1, 10 μM 24 h with 17-β-estradiol (E2). Cellular proteins were detected in total cell lysates by immunoblotting analysis with anti-BRK and anti-ERα antibodies and β-actin expression served as loading control. **Figure S3.** High BRK transcript level tends to correlate with poor ER+ breast cancer patient survival. Overall survival analysis of breast cancer patients’ samples from the TCGA data set: **A**) ER-positive versus all other subtypes combined (*n* = 1102; *p* = 6.3e-07). **B**) BRK expression significance in all subtypes combined (n = 1102; *p* = 0.65). **C**) BRK expression significance in ER-positive tumors (*n* = 491; *p* = 0.84). **D-G**) Effect of BRK expression on relapse-free survival (RFS), high (red) or low (black) BRK expressing, ER-positive breast cancer patient. **D**) Effect of BRK in ER-positive subtypes (hazard ration, HR = 1.14, *p* = 0.38). **E**) Effect of BRK in luminal A breast cancer patients (HR = 1.2, *p* = 0.37). **F**) Effect of BRK luminal B breast cancer patients (HR = 1.14, *p* = 0.13). **G**) RFS in tamoxifen-treatment patients. Note: Kaplan–Meier survival kmplots generated using the GSE1379 data set contains gene expression data from 60 ER+ patients treated with standard breast surgery and radiation followed by five years of systemic adjuvant tamoxifen. (PDF 328 kb)

## Abbreviations

BRK: Breast Tumor Kinase
ER: Estrogen Receptor
HER2: Human Epidermal growth factor Receptor 2
IDC: Invasive Ductal Carcinoma
IHC: Immunohistochemistry
LNM: Lymph Node Metastasis
PR: Progesterone Receptor
TMAs: Tissue Microarrays
TNBC: Triple-Negative Breast Cancer
