# Supplementary material for: Estrogen receptor signaling regulates the expression of the breast tumor kinase in breast cancer cells
Source: BMC Cancer. 2019 Jan 16;19:78. doi: 10.1186/s12885-018-5186-8 (PMC6335685; doi:10.1186/s12885-018-5186-8)
Supplement: Supplementary file 1 — Table S1. Differential expression of BRK mRNA in various cancers. Table S2. BRK mRNA expression in a TCGA cohort of breast cancer subtypes. Table S3. Invasive ductal carcinoma (IDC) samples, classified according to tumor grade. Clinical parameters for the 6 cases/24 cores array that contains 12 invasive ductal carcinoma (IDC) samples, classified according to tumor grade, and 12 adjacent normal mammary tissues. Table S4. Breast tumor samples classified according to lymph node metastasis ability. Clinical parameters for 50 cases/100 cores contained 50 cases of breast carcinoma (46 IDC, one micropapillary carcinoma, two invasive lobular carcinomas, and one neuroendocrine carcinoma) and 50 matched lymph node metastasis (LNM) samples. Table S5. Clinical and molecular characteristics of breast cancer and mammary epithelial cells. Classification of breast cancer cell lines as described by Neve et al. [22]. (PDF 404 kb) [file 12885_2018_5186_MOESM1_ESM.pdf]

Supplementary Table 1: Differential expression of BRK mRNA in various cancers

**Cancer**	**Sample size (Normal/tumor)**	**BRK expression in tumors relative to normal tissue**	**pValue**	**Significance**
BLCA (bladder urothelial carcinoma)	19/405	High	0.018	Significant
**BRCA (breast carcinoma)**	100/1084	High	$\mathbf{1.2 \times 10^{-31}}$	Significant
CESC (cervical squamous cell carcinoma)	3/297	High	0.012	Significant
CHOL (cholangiocarcinoma)	9/36	High	0.039	Significant
COAD (colon adenocarcinoma1)	41/476	Low	2.3×10^{-09}	Significant
ESCA (esophageal cancer)	11/173	High	0.276	Not significant
GBM (glioblastoma multiforme)	5/143	Low	0.023	Significant
HNSC (head and neck squamous cell carcinoma)	44/515	Low	3.0×10^{-09}	Significant
KICH (cervical squamous cell carcinoma)	25/66	Low	1.1×10^{-07}	Significant
KIRC (clear cell kidney carcinoma)	72/529	High	0.0004	Significant
KIRP (papillary kidney carcinoma)	32/289	High	0.0001	Significant
LIHC (liver hepatocellular carcinoma)	51/369	Low	0.0009	Significant
LUAD (lung adenocarcinoma)	58/512	High	1.1×10^{-15}	Significant
LUSC (lung squamous cell carcinoma)	51/498	High	7.5×10^{-06}	Significant
PAAD (pancreatic ductal carcinoma)	4/178	High	0.007	Significant
PCPG (pheochromocytoma and paraganglioma)	3/176	High	0.002	Significant
PRAD (prostate adenocarcinoma)	51/483	High	0.0001	Significant
READ (rectal adenocarcinoma)	10/163	High	0.1899	Not significant
SARC (sarcoma)	2/258	Inconclusive	0.305	Not significant
SKCM (cutaneous melanoma)	1/104	Inconclusive	0.153	Not significant
STAD (stomach adenocarcinoma)	35/413	Low	0.034	Significant
THCA (papillary thyroid carcinoma)	57/500	High	1.2×10^{-08}	Significant
THYM (thymoma)	2/120	Inconclusive	0.329	Not significant
UCEC (uterine corpus endometrial carcinoma)	35/540	High	6.0×10^{-09}	Significant

$p < 0.05$ = significant

Supplementary Table 2: BRK mRNA expression in a TCGA cohort of breast cancer subtypes

Subtype	No of patients in group		Significance against the normal tissue (pValue)
	High	Low	
Normal	114		-
Luminal	492		8.1×10^{-45}
HER2	39		2.3×10^{-11}
TNBC	152		0.002
Breast tumor	104		3.4×10^{-36}

Supplemetary Table 3

catalognum	position	age(41-52)	organ	pathology	grade	stage	tnm	type
BR243d	A1		Breast	Invasive ductal carcinoma	1	IIb	T2N1M0	Malignant
BR243d	A2		Breast	Invasive ductal carcinoma	1	IIb	T2N1M0	Malignant
BR243d	A3		Breast	Cancer adjacent normal breast tissue	-		-	NAT
BR243d	A4		Breast	Cancer adjacent normal breast tissue (fibrofatty tissue)	-		-	NAT
BR243d	A5		Breast	Invasive ductal carcinoma	1-2	IIb	T2N1M0	Malignant
BR243d	A6		Breast	Invasive ductal carcinoma	1-2	IIb	T2N1M0	Malignant
BR243d	A7		Breast	Cancer adjacent normal breast tissue	-		-	NAT
BR243d	A8		Breast	Cancer adjacent normal breast tissue	-		-	NAT
BR243d	B1		Breast	Invasive ductal carcinoma	2	IIIa	T2N2M0	Malignant
BR243d	B2		Breast	Invasive ductal carcinoma	2	IIIa	T2N2M0	Malignant
BR243d	B3		Breast	Cancer adjacent normal breast tissue	-		-	NAT
BR243d	B4		Breast	Cancer adjacent normal breast tissue	-		-	NAT
BR243d	B5		Breast	Invasive ductal carcinoma	2	IIa	T2N0M0	Malignant
BR243d	B6		Breast	Invasive ductal carcinoma	2	IIa	T2N0M0	Malignant
BR243d	B7		Breast	Cancer adjacent normal breast tissue (fibrofatty tissue)	-		-	NAT
BR243d	B8		Breast	Cancer adjacent normal breast tissue	-		-	NAT
BR243d	C1		Breast	Invasive ductal carcinoma	3	IIa	T2N0M0	Malignant
BR243d	C2		Breast	Invasive ductal carcinoma	3	IIa	T2N0M0	Malignant
BR243d	C3		Breast	Cancer adjacent normal breast tissue (adenosis)	-		-	NAT
BR243d	C4		Breast	Cancer adjacent normal breast tissue	-		-	NAT
BR243d	C5		Breast	Invasive ductal carcinoma	2-3	IIb	T3N1M0	Malignant
BR243d	C6		Breast	Invasive ductal carcinoma	3	IIb	T3N1M0	Malignant
BR243d	C7		Breast	Cancer adjacent normal breast tissue	-		-	NAT
BR243d	C8		Breast	Cancer adjacent normal breast tissue	-		-	NAT

Supplementary Table 4

catalognum	position	age (28-80) and Organ	pathology	grade	stage	tnm	er	pr	her2	type
BR10010a	A1	Breast	Invasive duct	1	IIIa	T2N2M0	-	-	3+	Malignant
BR10010a	A2	Breast	Invasive duct	1	IIa	T1N1M0	-	-	2+	Malignant
BR10010a	A3	Breast	Invasive duct	1	IIb	T2N1M0	+++, 95%	+++, 65%	0	Malignant
BR10010a	A4	Breast	Invasive duct	1	IIb	T2N1M0	+, 35%	++, 60%	0	Malignant
BR10010a	A5	Breast	Invasive duct	1	IIb	T3N1M0	-	-	0	Malignant
BR10010a	A6	Breast	Invasive duct	2	IIb	T2N1M0	-	-	3+	Malignant
BR10010a	A7	Breast	Invasive duct	2	IIIa	T2N2M0	++, 95%	++, 80%	1+	Malignant
BR10010a	A8	Breast	Invasive duct	2	IIb	T2N1M0	-	+, 45%	2+	Malignant
BR10010a	A9	Breast	Invasive duct	2	IIb	T2N1M0	+++, 90%	+++, 70%	0	Malignant
BR10010a	A10	Breast	Invasive duct	2	IIb	T2N1M0	-	-	0	Malignant
BR10010a	B1	Breast	Invasive duct	2	IIIa	T2N2M0	+, 15%	+, 5%	0	Malignant
BR10010a	B2	Breast	Invasive duct	2	IIb	T2N1M0	-	-	0	Malignant
BR10010a	B3	Breast	Invasive duct	2	IIb	T2N1M0	-	+, 40%	0	Malignant
BR10010a	B4	Breast	Invasive duct	2	IIIb	T4N2M0	+, 20%	+++, 99%	0	Malignant
BR10010a	B5	Breast	Invasive duct	2	IIIa	T2N2M0	-	-	3+	Malignant
BR10010a	B6	Breast	Invasive duct	2	IIb	T2N1M0	-	-	3+	Malignant
BR10010a	B7	Breast	Invasive duct	2	IIIa	T2N2M0	-	+, 10%	0	Malignant
BR10010a	B8	Breast	Invasive duct	2	IIb	T3N1M0	-	-	3+	Malignant
BR10010a	B9	Breast	Invasive duct	2	IIb	T2N1M0	-	-	2+	Malignant
BR10010a	B10	Breast	Invasive duct	2	IIb	T2N1M0	-	-	0	Malignant
BR10010a	C1	Breast	Invasive duct	2	IIIa	T2N2M0	-	++, 55%	1+	Malignant
BR10010a	C2	Breast	Invasive duct	2	IIb	T3N1M0	+, 20%	+++, 10%	0	Malignant
BR10010a	C3	Breast	Invasive duct	1	IIb	T2N1M0	-	-	3+	Malignant
BR10010a	C4	Breast	Invasive duct	2	IIb	T2N1M0	-	-	0	Malignant
BR10010a	C5	Breast	Invasive duct	2	IIIa	T2N2M0	-	-	1+	Malignant
BR10010a	C6	Breast	Invasive duct	2	IIIa	T3N2M0	-	-	2+	Malignant
BR10010a	C7	Breast	Invasive duct	2	IIIa	T2N2M0	+, 10%	-	3+	Malignant
BR10010a	C8	Breast	Invasive duct	2	IIIb	T4N1M0	+, 5%	-	3+	Malignant
BR10010a	C9	Breast	Invasive duct	2	IIIa	T2N2M0	+, 80%	++, 8%	0	Malignant
BR10010a	C10	Breast	Invasive duct	2	IIb	T2N1M0	-	-	0	Malignant
BR10010a	D1	Breast	Invasive duct	2	IIb	T2N1M0	-	-	0	Malignant
BR10010a	D2	Breast	Invasive duct	2	IIb	T2N1M0	+++, 95%	+++, 50%	0	Malignant
BR10010a	D3	Breast	Invasive duct	3	IIb	T3N1M0	+, 10%	-	0	Malignant
BR10010a	D4	Breast	Invasive duct	2	IIb	T2N1M0	++, 90%	-	2+	Malignant
BR10010a	D5	Breast	Invasive duct	2	IIb	T3N1M0	-	-	0	Malignant
BR10010a	D6	Breast	Invasive duct	2	IIIa	T3N2M0	+, 8%	-	1+	Malignant
BR10010a	D7	Breast	Invasive duct	2	IIb	T3N1M0	++, 25%	-	3+	Malignant
BR10010a	D8	Breast	Invasive duct	2	IIb	T2N1M0	-	-	0	Malignant
BR10010a	D9	Breast	Invasive duct	2	IIb	T2N1M0	-	++, 70%	0	Malignant
BR10010a	D10	Breast	Invasive duct	2	IIIa	T2N2M0	-	-	3+	Malignant
BR10010a	E1	Breast	Invasive duct	2	IIb	T3N1M0	+, 5%	++, 8%	0	Malignant
BR10010a	E2	Breast	Invasive duct	2	IIIa	T2N2M0	+, 5%	+++, 95%	0	Malignant
BR10010a	E3	Breast	Invasive duct	3	IIb	T3N1M0	-	-	0	Malignant
BR10010a	E4	Breast	Invasive duct	2	IIIb	T4N2M0	-	-	1+	Malignant
BR10010a	E5	Breast	Invasive duct	3	IIb	T2N1M0	-	-	0	Malignant
BR10010a	E6	Breast	Invasive duct	3	IIb	T2N1M0	-	-	3+	Malignant
BR10010a	E7	Breast	Invasive micr	-	IIb	T2N1M0	-	-	3+	Malignant
BR10010a	E8	Breast	Invasive lobu	-	IIb	T2N1M0	-	-	0	Malignant
BR10010a	E9	Breast	Invasive lobu	-	IIb	T2N1M0	-	-	0	Malignant
BR10010a	E10	Breast	Neuroendocr	-	IIb	T2N1M0	+++, 45%	-	1+	Malignant
BR10010a	F1	Lymph node	Metastatic carcinoma from breast (lymph node tissue)	-				- -	- 0-	Metastasis
BR10010a	F2	Lymph node	Metastatic carcinoma from breast	-		- -	-	-	3+	Metastasis
BR10010a	F3	Lymph node	Metastatic carcinoma from breast	-		-	++, 100%	++, 100%	0	Metastasis
BR10010a	F4	Lymph node	Metastatic carcinoma from breast	-		-	-	++, 90%	0	Metastasis
BR10010a	F5	Lymph node	Metastatic carcinoma from breast	-		-	-	-	0	Metastasis
BR10010a	F6	Lymph node	Metastatic carcinoma from breast	-		-	-	-	3+	Metastasis
BR10010a	F7	Lymph node	Metastatic carcinoma from breast	-		-	+, 80%	-	2+	Metastasis
BR10010a	F8	Lymph node	Metastatic carcinoma from breast	-		- -	-	+++, 45%	2+	Metastasis
BR10010a	F9	Lymph node	Metastatic carcinoma from breast	-		-	+++,-95%	+++, 99%	0	Metastasis
BR10010a	F10	Lymph node	Metastatic carcinoma from breast	-		-	-	-	0	Metastasis
BR10010a	G1	Lymph node	Metastatic carcinoma from breast	-		-	-	-	1+	Metastasis
BR10010a	G2	Lymph node	Metastatic carcinoma from breast	-		-	++, 95%	+++, 99%	0	Metastasis
BR10010a	G3	Lymph node	Metastatic carcinoma from breast	-		- -	-	-	3+	Metastasis
BR10010a	G4	Lymph node	Metastatic carcinoma from breast	-			++, 80%	+++, 70%	0	Metastasis
BR10010a	G5	Lymph node	Metastatic carcinoma from breast	-		-	-	-	3+	Metastasis
BR10010a	G6	Lymph node	Metastatic carcinoma from breast	-		-	-	-	3+	Metastasis
BR10010a	G7	Lymph node	Metastatic carcinoma from breast	-		-	-	++, 40%	0	Metastasis
BR10010a	G8	Lymph node	Metastatic carcinoma from breast	-		-	-	-	3+	Metastasis
BR10010a	G9	Lymph node	Metastatic carcinoma from breast	-		-	-	-	3+	Metastasis
BR10010a	G10	Lymph node	Metastatic carcinoma from breast	-		-	+, 10%	+, 5%	0	Metastasis
BR10010a	H1	Lymph node	Metastatic carcinoma from breast	-		- -	-	++, 80%	1+	Metastasis
BR10010a	H2	Lymph node	Metastatic carcinoma from breast	-		-	+++,-90%	-	0	Metastasis
BR10010a	H3	Lymph node	Metastatic carcinoma from breast	-		-	-	-	3+	Metastasis
BR10010a	H4	Lymph node	Metastatic carcinoma from breast	-		-	-	+, 5%	0	Metastasis
BR10010a	H5	Lymph node	Metastatic carcinoma from breast	-		-	-	-	0	Metastasis
BR10010a	H6	Lymph node	Metastatic carcinoma from breast	-		-	-	-	3+	Metastasis
BR10010a	H7	Lymph node	Metastatic carcinoma from breast	-		-	-	-	3+	Metastasis
BR10010a	H8	Lymph node	Metastatic carcinoma from breast	-		-	-	-	3+	Metastasis
BR10010a	H9	Lymph node	Metastatic carcinoma from breast	-		-	+, 30%	+++, 95%	0	Metastasis
BR10010a	H10	Lymph node	Metastatic carcinoma from breast	-		-	-	-	0	Metastasis
BR10010a	I1	Lymph node	Metastatic carcinoma from breast	-		-	-	-	0	Metastasis
BR10010a	I2	Lymph node	Metastatic carcinoma from breast	-		-	++, 25%	-	3+	Metastasis
BR10010a	I3	Lymph node	Metastatic carcinoma from breast	-		- -	-	-	0	Metastasis
BR10010a	I4	Lymph node	Metastatic carcinoma from breast	-			+, 15%	+++, 5%	2+	Metastasis
BR10010a	I5	Lymph node	Metastatic carcinoma from breast	-			+, 15%	+, 5%	0	Metastasis
BR10010a	I6	Lymph node	Metastatic carcinoma from breast	-		- -	-	-	1+	Metastasis
BR10010a	I7	Lymph node	Metastatic carcinoma from breast	-		-	+, 20%	-	3+	Metastasis
BR10010a	I8	Lymph node	Metastatic carcinoma from breast	-		-	-	-	0	Metastasis
BR10010a	I9	Lymph node	Metastatic carcinoma from breast	-		-	-	+, 5%	0	Metastasis
BR10010a	I10	Lymph node	Metastatic carcinoma from breast	-		-	-	-	3+	Metastasis
BR10010a	J1	Lymph node	Metastatic carcinoma from breast	-		-	+, 20%	++, 3%	0	Metastasis
BR10010a	J2	Lymph node	Metastatic carcinoma from breast	-		-	-	+, 3%	0	Metastasis
BR10010a	J3	Lymph node	Metastatic carcinoma from breast	-		-	-	-	1+	Metastasis
BR10010a	J4	Lymph node	Metastatic carcinoma from breast	-		-	-	-	0	Metastasis
BR10010a	J5	Lymph node	Metastatic carcinoma from breast	-		-	-	-	0	Metastasis
BR10010a	J6	Lymph node	Metastatic carcinoma from breast (sparse)	-			–	–	2+	Metastasis
BR10010a	J7	Lymph node	Metastatic carcinoma from breast	-		-	-	-	2+	Metastasis
BR10010a	J8	Lymph node	Metastatic carcinoma from breast	-		-	-	-	0	Metastasis
BR10010a	J9	Lymph node	Metastatic carcinoma from breast	-		-	-	-	0	Metastasis
BR10010a	J10	Lymph node	Metastatic carcinoma from breast	-		-	+++,-90%	-	1+	Metastasis

Supplementary Table 5: Clinical and molecular characteristics of breast cancer cells and mammary epithelial cells.

Cell line		[a]Gene cluster	[a]Derived Tumor type	[a]Tumo-rigenic	[a]ER	[a]PR	[a]HER2
1	184B5	NA	N, MG	No	NA	NA	NA
2	AU565a	Luminal	AC	NA	-	-	+
3	BT20	Basal A	IDC	Yes	-	-	-
4	BT474	Luminal	IDC	Yes	+	+	+
5	BT483	Luminal	IDC	Yes	+	+	-
6	BT549	Basal B	IDC	Yes	-	-	-
7	CAMA1	Luminal	AC	Yes	+	-	-
8	DU4475	NA	IDC	Yes	-	-	-
9	HCC38	Basal B	DCIS	NA	-	-	-
10	HCC70	Basal A	DCIS	NA	+	-	-
11	HCC202	Luminal	DCIS	NA	-	-	+
12	HCC1187	Basal A	DCIS		-	-	-
13	HCC1395	Basal B	DCIS		-	-	-
14	HCC1419	Luminal	DCIS		-	-	+
15	HCC1428	Luminal	Met adenoca		+	+	-
16	HCC1500	Basal B	DCIS	NA	-	-	-
17	HCC1569	Basal A	MC	NA	-	-	+
18	HCC1599	Basal A	DCIS		-	-	-
19	HCC1806	NA	ASC		-	-	-
20	HCC1937	Basal A	DCIS	NA	-	-	-
21	HCC1954	Basal A	DCIS		NA	NA	NA
22	HCC2157	Basal A	DCIS		-	+	+
23	HCC2218	Luminal	MLCa		-	-	+
24	HS578T	Basal B	IDC	No	-	-	-
25	MCF7	Luminal	IDC	Yes	+	+	-
26	MCF10A	Basal B	F	No	-	-	-
27	MCF10F	Basal B	F	No	-	-	-
28	MCF12A	Basal B	F	No	-	-	-
29	[b]MDAKb2	Luminal	AC	No	-	-	-
30	MDA-MB-134VI	Luminal	IDC	Yes	+	-	-
31	MDA-MB-157	Basal B	MC	Yes	-	-	-
32	MDA-MB-175VII	Luminal	IDC	Yes	+	-	-
33	MDA-MB-231	Basal B	AC	Yes	-	-	+
34	MDA-MB-361	Luminal	AC	Yes	+	-	-
35	MDA-MB-415	Luminal	AC	No	+	-	-
36	MDA-MB-436	Basal B	IDC	No	-	-	-
37	MDA-MB-453	Luminal	AC	No	-	-	-
38	MDA-MB-468	Basal A	AC	Yes	-	-	-
39	SKBR3	Luminal	AC	Yes	-	-	+
40	T47D	Luminal	AnCa	Yes	+	+	+
41	UACC812	Luminal	IDC	NA	+	-	-
42	UACC893	Luminal	IDC	NA	-	-	
43	ZR751	Luminal	IDC	Yes	+	-	-
44	ZR7530	Luminal	IDC	NA	+	-	+
45	MDA-MB-435	Basal B	IDC	NA	-	-	-
46	HBL100	Basal B	N, BM	Yes	+	-	-

Supplementary Table 5

AC, adenocarcinoma; AnCa, anaplastic carcinoma; ASC, acantholytic squamous carcinoma; BM, breast milk; DCIS, ductal carcinoma in situ; F, fibrocystic disease; IDC, invasive ductal carcinoma; MC, metaplastic carcinoma; Met adenoca, Metastatic adenocarcinoma; MG, mammargy gland; MLCa, metastatic lobular carcinoma; N, normal (non-tumor-derived cells); NA, Not available, undetermined or unknown events.
The highest expression of BRK occurs in IDC in bold.
No expression is indicated by (-) and positive expression by (+).
Discrepancies in expression are denoted by (+/-).

[a]These specifications were compiled using published data from Neve *et al*. [22] and Gazdar *et al*. [23] and also from information provided by ATCC for all cell lines except for HBL100 whose specifications came from Cell Lines Services. Additional information on DU4475 and the HCC series of cell lines came from Langlois *et al.* [24] and Kao *et al.* [25].

[b]MDAKb2 was derived from MDA-MB-453 cells stably transformed with the MMTV.luciferase.neo reporter gene construct by Wilson *et al.* [26].
