# Supplementary figures and images for: Estrogen receptor signaling regulates the expression of the breast tumor kinase in breast cancer cells
Source: BMC Cancer. 2019 Jan 16;19:78. doi: 10.1186/s12885-018-5186-8 (PMC6335685; doi:10.1186/s12885-018-5186-8)

# Supplementary Figure 1

A

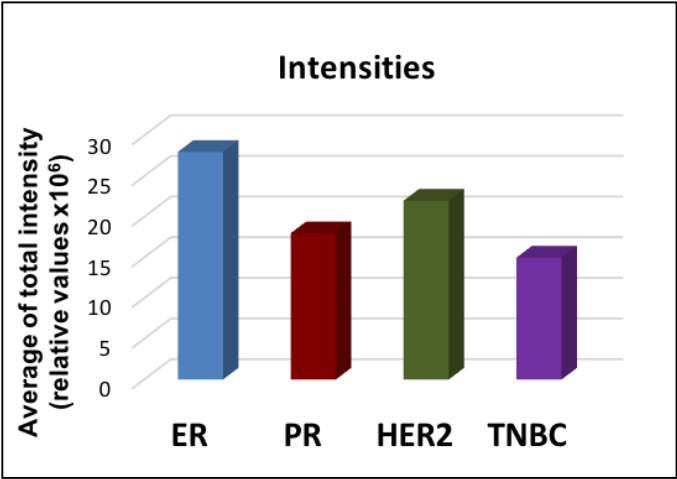

B

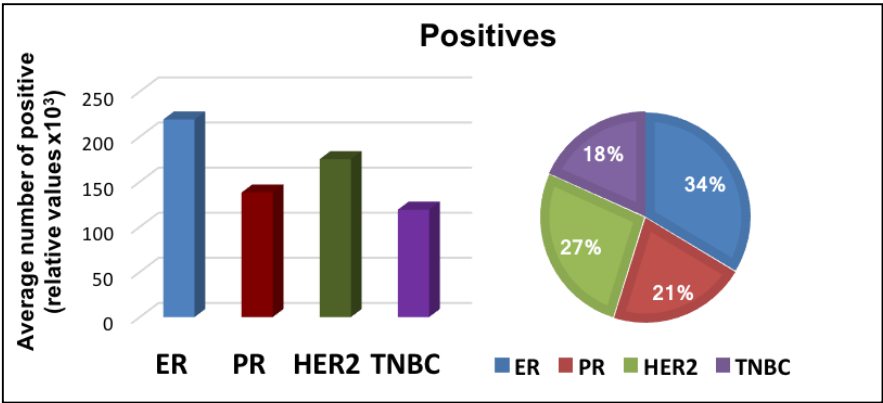

**Supplementary Figure 2**

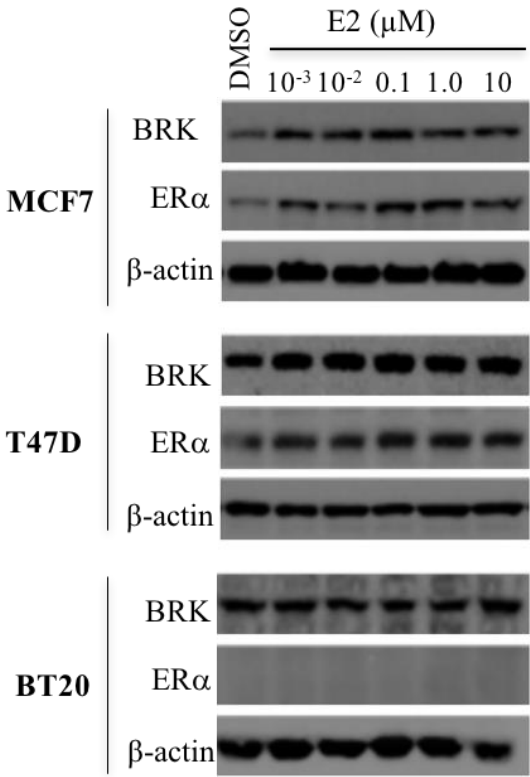

# Supplementary Figure 3

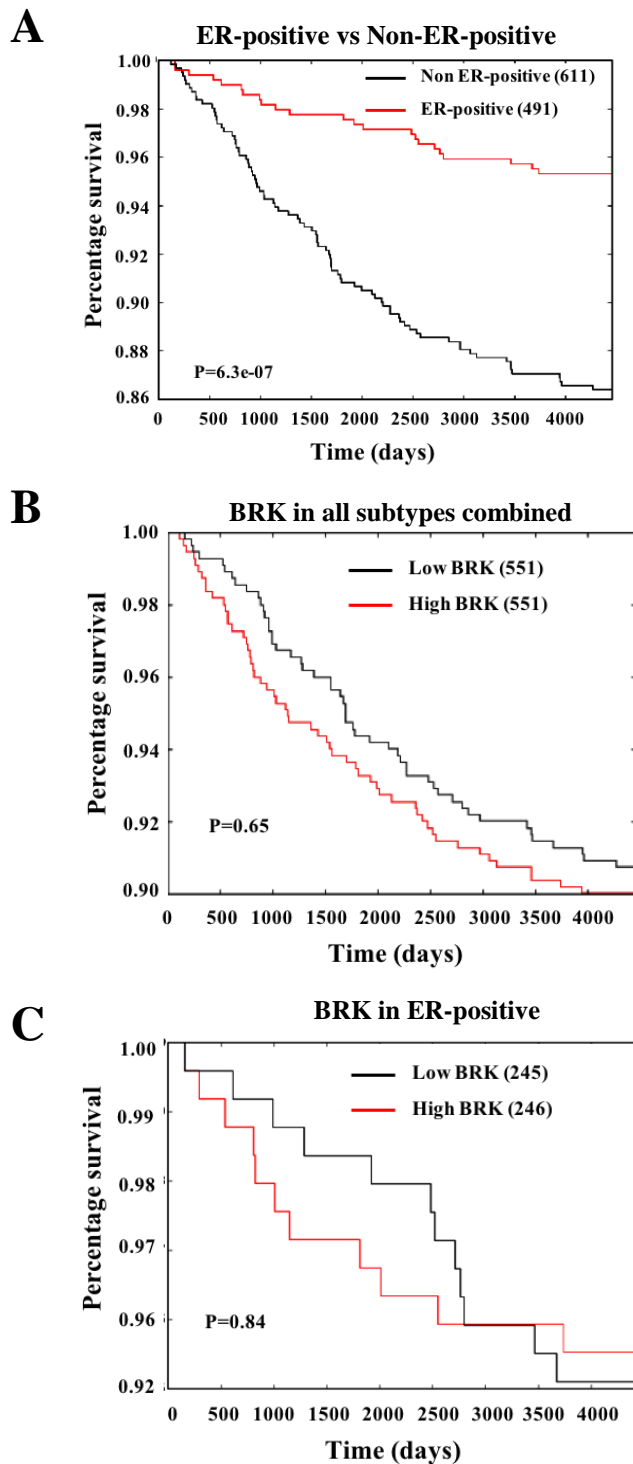

# Supplementary Figure 3

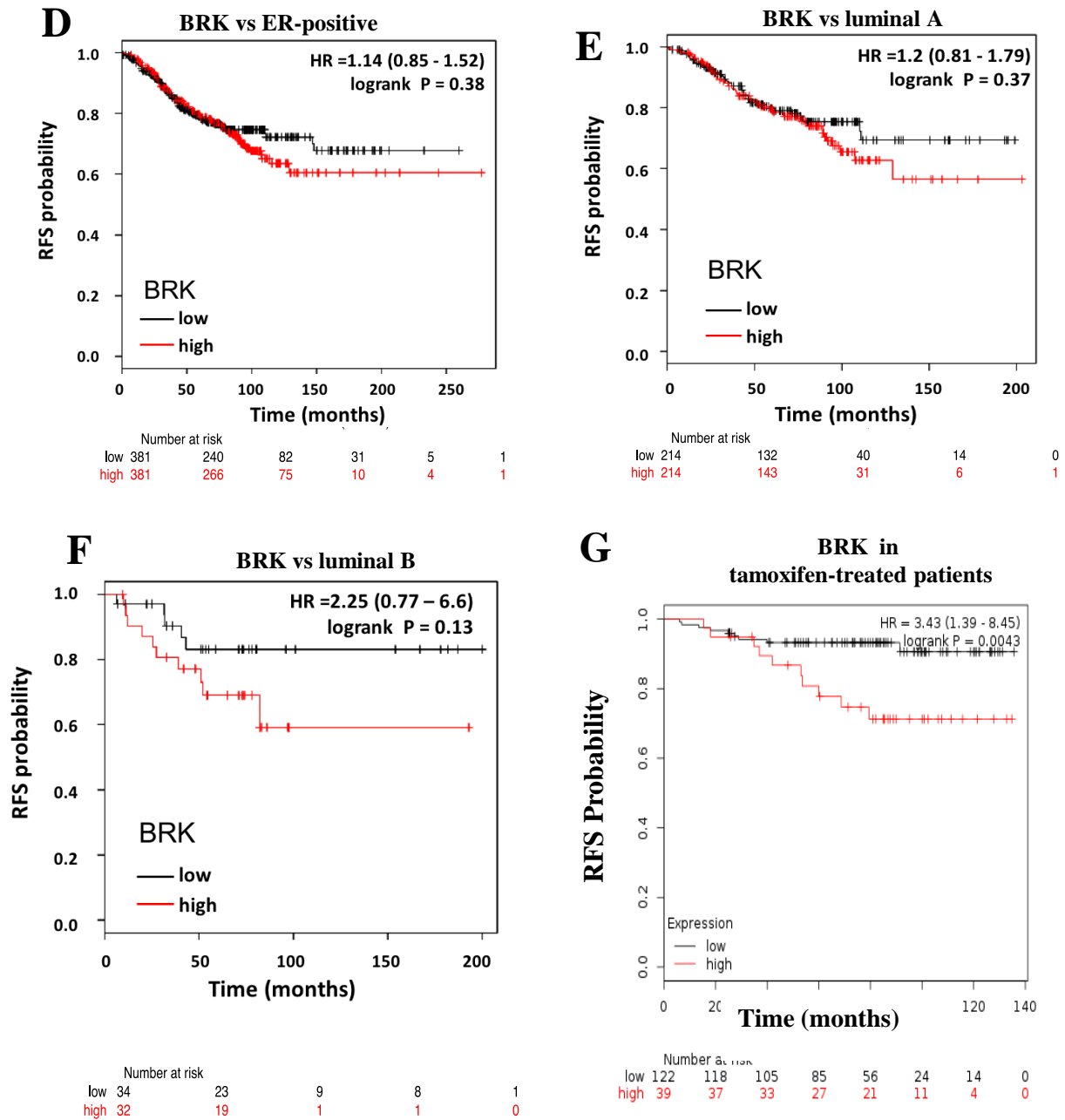

Supplement: Supplementary file 2 — Figure S1. Molecular subtype of clinical tumor tissues. The absolute values for the total intensity and total number of positive BRK staining for each sample in the 50 cases/100 cores array (BR10010a, USBIOMAX, USA) were provided by the pathologists at USBIOMAX. Based on the clinical information provided, the samples were grouped into their respective molecular subtype: ER, PR, HER2, and triple negative. The average total intensities and number of positives for each subtype were calculated and plotted on the graphs. A) Average total intensity per subtype. B) Average total number of positive per subtype. Figure S2. Estradiol dose dependent BRK and ERα protein expression in breast cancer cell lines. MCF7, T47D and BT20 cells were treated with 0.001, 0.01, 0.1, 1, 10 μM 24 h with 17-β-estradiol (E2). Cellular proteins were detected in total cell lysates by immunoblotting analysis with anti-BRK and anti-ERα antibodies and β-actin expression served as loading control. Figure S3. High BRK transcript level tends to correlate with poor ER+ breast cancer patient survival. Overall survival analysis of breast cancer patients’ samples from the TCGA data set: A) ER-positive versus all other subtypes combined (n = 1102; p = 6.3e-07). B) BRK expression significance in all subtypes combined (n = 1102; p = 0.65). C) BRK expression significance in ER-positive tumors (n = 491; p = 0.84). D-G) Effect of BRK expression on relapse-free survival (RFS), high (red) or low (black) BRK expressing, ER-positive breast cancer patient. D) Effect of BRK in ER-positive subtypes (hazard ration, HR = 1.14, p = 0.38). E) Effect of BRK in luminal A breast cancer patients (HR = 1.2, p = 0.37). F) Effect of BRK luminal B breast cancer patients (HR = 1.14, p = 0.13). G) RFS in tamoxifen-treatment patients. Note: Kaplan–Meier survival kmplots generated using the GSE1379 data set contains gene expression data from 60 ER+ patients treated with standard breast surgery and radiation followed by five years of s [file 12885_2018_5186_MOESM2_ESM.pdf]
